# Influence of lung aeration on diaphragmatic contractility during a spontaneous breathing trial: an ultrasound study

**DOI:** 10.1186/s40560-019-0409-x

**Published:** 2019-12-02

**Authors:** Jing Xia, Chuan-Yun Qian, Li Yang, Mei-Ju Li, Xiao-Xue Liu, Ting Yang, Qin Lu

**Affiliations:** 1 Emergency Department, The First Affiliated Hospital of Kunming Medical University, 295 Xi Chang Road, Kunming, 650032 Yunnan China; 2 Multidisciplinary Intensive Care Unit, Department of Anesthesiology and Critical Care, La Pitié-Salpêtrière Hospital, Assistance Publique-Hôpitaux de Paris, Sorbonne Université, Paris, France

**Keywords:** Spontaneous breathing trial, Ultrasound, Lung aeration, Diaphragmatic thickening fraction, Weaning, Mechanical ventilation

## Abstract

**Background:**

A spontaneous breathing trial (SBT) is a major diagnostic tool to predict successfully extubation in patients. Several factors may lead to weaning failure, including the degree of lung aeration loss and diaphragm dysfunction. The main objective was to compare the diaphragmatic contractility between patients with high lung aeration loss and low lung aeration loss during a 30-minute SBT by ultrasound.

**Methods:**

This was a prospective single-center study. Lung ultrasound aeration score (LUS) and diaphragmatic thickening fraction (DTF) were measured during mechanical ventilation 1 h before SBT (T-1), 30 min (T1), and 120 min (T2) after the start of the SBT during quiet breathing. The right and left DTF were compared between patients with LUS ≥ 14 (high lung aeration loss), considered at high risk of post-extubation distress, and those with LUS < 14 (low lung aeration loss). The relationship between the LUS and DTF and the changes in LUS and DTF from T-1 to T2 in patients with LUS ≥ 14 were assessed.

**Results:**

Forty-nine patients were analyzed; 33 had LUS ≥ 14 and 16 had LUS < 14 at T1. The DTF at T1 was significantly higher in patients with LUS ≥ 14 than in those with LUS < 14: the right median (IQR) DTF was 22.2% (17.1 to 30.9%) vs. 14.8% (10.2 to 27.0%) (*p* = 0.035), and the left median (IQR) DTF was 25.0% (18.4 to 35.0%) vs. 18.6% (9.7 to 24.2%) (*p* = 0.017), respectively. There was a moderate positive correlation between the LUS and the DTF (Rho = 0.3, *p* = 0.014). A significant increase in the LUS was observed from T-1 to T1, whereas no change was found between T1 and T2. The DTF remained stable from T-1 to T2.

**Conclusions:**

During a SBT, diaphragmatic contraction acts differently depending on the degree of pulmonary aeration. In patients with high lung aeration loss, increased diaphragmatic contractility indicates an additional respiratory effort to compensate lung volume loss that would contribute to successful SBT. Further studies are needed to evaluate the combined evaluation of lung aeration and diaphragmatic function to predict the successful weaning of patients from mechanical ventilation.

## Background

A spontaneous breathing trial (SBT) is a major diagnostic tool to determine whether patients can be successfully extubated [1, 2]. A number of factors may be responsible for the failure of a SBT and/or post-extubation distress, such as cardiovascular diseases, lung aeration loss [3–5], and dysfunction of the respiratory muscles, including diaphragmatic dysfunction [6, 7].

In patients with difficult weaning, significant decreases in tidal variation of impedance and end-expiratory lung volume were observed after patient ventilator disconnection during a SBT [8]. Similarly, the loss of lung aeration at the end of a SBT assessed by ultrasonography was significantly greater in patients who developed post-extubation distress than in those who were definitively weaned from mechanical ventilation [4]. Using the lung aeration ultrasound score (LUS), a cutoff value of 14 with an area under the curve of 0.86 was predictive of patients at high risk of post-extubation distress [4]. On the other hand, in patients with prolonged mechanical ventilation, it has been shown that the diaphragmatic thickening fraction (DTF), defined as the diaphragmatic thickness at end-inspiration (TEI) minus the diaphragmatic thickness at end-expiration (TEE) divided by the TEE, was significantly lower in patients who failed the SBT than in patients who had a successful SBT [9].

Lung volume and diaphragmatic function are strongly related. Inspired volume is known to correlate linearly and positively with diaphragmatic excursion [10, 11]. As the lung volume increases from functional residual capacity (FRC) to total lung capacity (TLC), the diaphragmatic thickness increases significantly [12, 13]. In addition, in mechanically ventilated patients, the DTF decreased when the level of pressure support increased, suggesting that an increase in lung volume resulting from a relatively high level of pressure support could reduce the diaphragmatic workload [14]. In patients under mechanical ventilation, diaphragmatic dysfunction is frequently observed due to sepsis, neuropathy, or muscular atrophy [5]. Consequently, the relationship between diaphragm contractile activity and pulmonary aeration becomes complex, and the capacity of diaphragm to compensate lung volume loss during a SBT remains unknown. To our knowledge, the impact of lung aeration loss on diaphragmatic contractility has not been evaluated by ultrasound.

Thus, the primary objective of the study was to compare the DTF between patients with high lung aeration loss (LUS ≥ 14) and those with low lung aeration loss (LUS < 14) during a 30-min SBT. The secondary objectives were to assess the correlation between the LUS and DTF, as well as the changes in diaphragm function and lung aeration before and after ventilator disconnection for a SBT in patients with LUS ≥ 14.

## Methods

### Patients and study protocol

This was a prospective single-center cohort study. Patients were recruited between March 2018 and May 2019 from the Intensive Care Unit (ICU) of a university hospital. This study was approved by the Ethical Institutional Review Board of Kunming Medical University, and written informed consent of patients and/or their relatives was obtained before inclusion. Inclusion criteria were patients aged ≥ 18 years, required invasive mechanical ventilation for ≥ 48 h and fulfilled the conditions for the first SBT [4]. Exclusion criteria were patients (1) underwent a tracheostomy, (2) presented paraplegia with medullar level above C6, (3) had chronic heart disease or severe left cardiac dysfunction (ejection fraction < 30%) before the SBT, (4) had severe neuromyopathy, (5) experienced SBT failure before the 30-min SBT or unplanned self-extubation before the SBT, and (6) had a poor acoustic window.

When intubated and mechanically ventilated patients fulfilled the conditions [9, 15] for a SBT, a trial of 120 min through a T-tube was performed as previously described [15]. Oxygen flow of 5 to 9 L/min was delivered through the T-tube during the SBT. The conditions for SBT failure were defined as a patient presenting at least 2 of the following criteria [3, 4]: (1) respiratory rate > 30/min, (2) use of accessory inspiratory muscles, (3) SpO_2_ < 90% with O_2_ > 9 L/min; (4) heart rate > 120/min or variation > 20%, (5) systolic arterial pressure > 200 mmHg or < 90 mmHg, (6) presence of delirium, sweating, drowsiness, hypercapnic, or hypoxic encephalopathy, (7) major bronchial obstruction at the end of the SBT with insufficient cough, and (8) hypercapnia > 50 mmHg or pH < 7.35 with an increase in PaCO_2_ > 10 mmHg.

Each subject was assessed by transthoracic lung ultrasound and diaphragmatic ultrasound during mechanical ventilation 1 h before the SBT (T-1), 30 min after the start of SBT (T1) and 120 min after the start of SBT (T2). Transthoracic lung ultrasound and diaphragmatic ultrasound were performed by two physicians (JX and LY) who had completed ultrasound training. Blood gases were analyzed, and vital signs were recorded at the same time points.

### Lung ultrasound

**Fig. 1:**
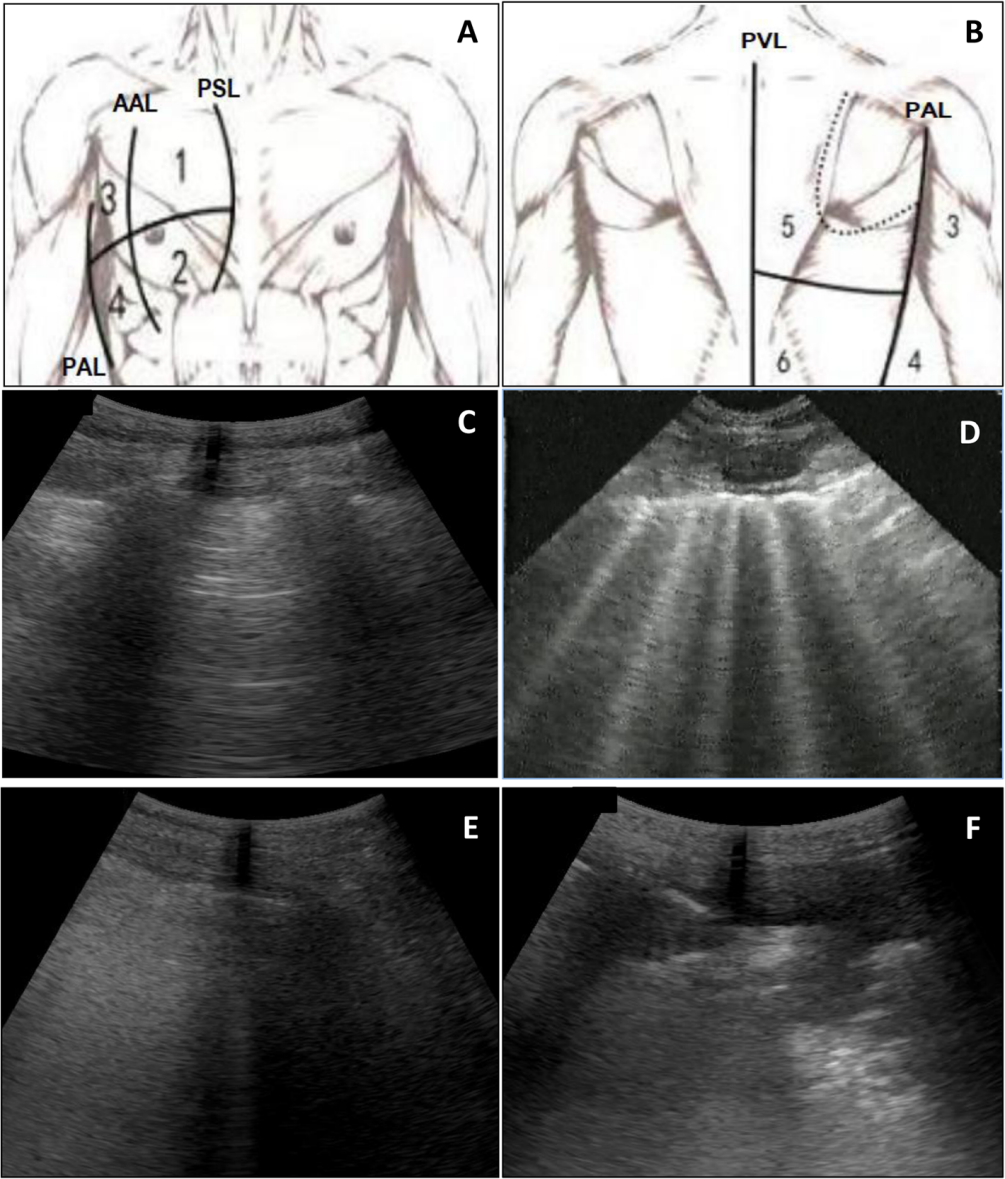
Ultrasound assessment of lung aeration score (LUS). Images **a** and **b** show 6 lung regions of the right lung; antero-superior (1), antero-inferior (2), latero-superior (3), latero-inferior (4), postero-superior (5), and posterior-inferior (6). Lung areas are delineated by parasternal (PSL), anterior axillary (AAL), posterior axillary (PAL), and paravertebral (PVL) lines. A numeric value was assigned to each lung area according to the most severe lung ultrasound finding. Images **c**, **d**, **e,** and **f** show typical images corresponding respectively to the degree of lung aeration loss. Image **c** shows normal lung image with presence of horizontal A lines (numeric value = 0); Image **d** shows the presence of multiple well-defined and spaced B1 lines issued from a small juxtapleural consolidation (numeric value = 1); image **e** shows multiple coalescent vertical B2 lines issued from the pleural line (numeric value = 2); image **f** shows the presence of tissue pattern containing either hyperechoic punctiform or linear images (numeric value = 3). LUS is calculated as the sum of the numeric values assigned to 12 lung zones ranging from 0 and 36

A GE Logiq E ultrasound device (General Electric Medical system, Wisconsin, USA) equipped with a 2–5 MHz convex probe was used for lung aeration assessment. In each subject, upper and lower lung areas of the right and left lungs were delineated by the parasternal, anterior axillary, and posterior axillary and paravertebral lines. Therefore, 12 lung regions corresponding to antero-superior, antero-inferior, latero-superior, latero-inferior, postero-superior, and posterior-inferior lung areas were examined [4]. A numeric value was assigned to each area according to the most severe lung ultrasound finding detected in the corresponding intercostal space as follows: 0 = normal aeration, defined by the presence of lung sliding with horizontal A lines or fewer than 2 isolated vertical B lines; 1 = moderate loss of lung aeration, defined as the presence of either multiple well-defined and spaced B1 lines issued from the pleural line or from small juxtapleural consolidations and corresponding to interstitial edema, or coalescent B1 lines issued from the pleural line or from small juxtapleural consolidations present in a limited portion of the intercostal space, corresponding to localized alveolar edema; 2 = severe loss of lung aeration: multiple coalescent vertical B2 lines issued from either the pleural line or from juxtapleural consolidations and detected in the whole area of one or several intercostal spaces, which correspond to diffuse alveolar edema; and 3 = lung consolidation, defined as the presence of a tissue pattern that contains either hyperechoic punctiform or linear images representative of static air bronchograms or the same images with inspiratory centrifugal movement, representative of dynamic air bronchograms [16], which correspond to complete loss of aeration. The LUS was calculated as the sum of the numeric values assigned to each lung zone, ranging from 0 to 36 (Fig. 1) [17].

### Diaphragm ultrasound

#### Diaphragmatic excursion

Right diaphragmatic excursion (DE) was measured with a 2–4 MHz probe during quiet breathing. Patients were in a semirecumbent position at an angle between 30 and 45° for the duration of the examination [18, 19]. The probe was positioned below the right costal margin craniocaudally in the midclavicular line; the probe was directed medially, cephalad, and dorsally so that the beam perpendicularly reached the posterior third of the corresponding hemidiaphragm as previously described [18]. B-mode was initially used to select the exploration line, and then M-mode was applied to display the motion of the diaphragm along the selected line [13, 18].

### Diaphragmatic thickness and diaphragmatic thickening fraction

**Fig. 2:**
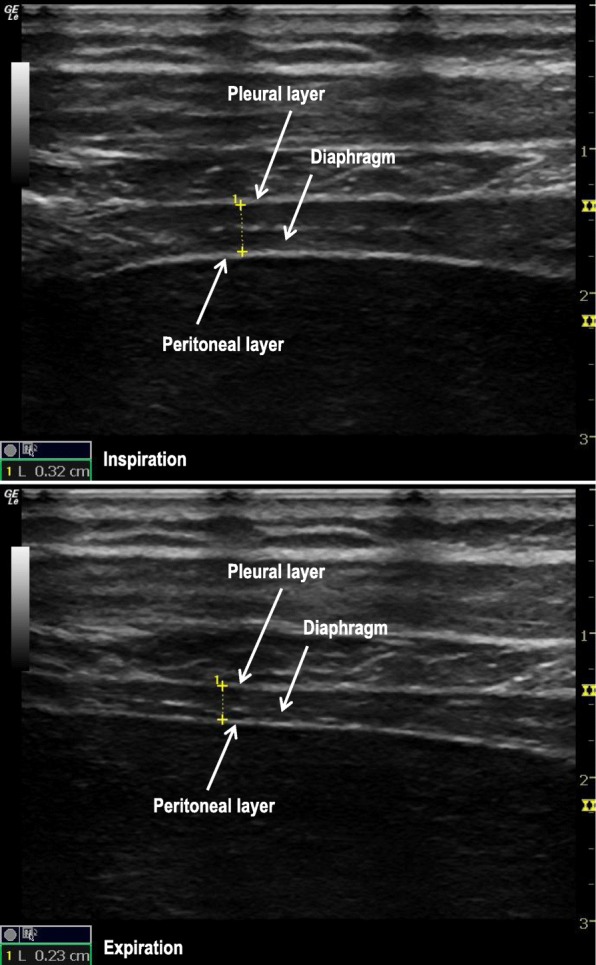
Ultrasound assessment of diaphragmatic thickness and diaphragmatic thickening fraction (DTF). Diaphragmatic thickness was assessed in B-mode at end-inspiration (upper panel) and end-expiration (lower panel) during quiet breathing. The white arrows indicate pleural layer and peritoneal layer. Diaphragm is between the 2 layers. The dashed line between the 2 crosses indicates diaphragm thickness; its value is shown at left lower corner of the image. DTF is defined as the diaphragmatic thickness at end-inspiration minus the diaphragmatic thickness at end-expiration (TEE) divided by the TEE, expressed as percentage

Diaphragmatic thickness measurements were performed with a 7–12 MHz linear probe. The probe was positioned in the apposition zone (corresponding to the area of the chest wall where the abdominal contents reach the lower rib cage) at the 8–10 intercostal space near the middle-axillary and angled perpendicular to the chest wall. In this location, the diaphragm was identified as a three-layered structure consisting of a relatively nonechogenic muscular layer bounded by the echogenic membranes of the diaphragmatic pleura and peritoneum. In the B-mode image, right and left diaphragmatic thickness, defined as the distance between the two membranes, were measured from the middle of the pleural membrane to the middle of the peritoneal membrane at end-expiration and end-inspiration during quiet breathing (Fig. 2) [13].

Measurements of diaphragmatic TEI and diaphragmatic TEE needed to match in simultaneously respiratory cycles. When breathing movement was not displayed on the screen, the TEE was measured just before the start of thickening (minimal thickness), and the TEI was measured at the maximal thickness recorded at the end of each period. Three consecutive measurements were averaged. The placement site of the probe was marked during the first measurement, which served as a reference site for the following measurements.

DTF was defined as the TEI minus the TEE divided by the TEE and was expressed as a percentage [14].

### Data collection

The following data were collected for each patient: age, sex, Sepsis-related Organ Failure Assessment score, Acute Physiology and Chronic Health Evaluation II score at ICU admission and clinical and ventilator parameters at inclusion. The duration of mechanical ventilation before the SBT was also recorded. The LUS of right and left lungs, total LUS score, right DE, right and left TEE and TEI, right and left DTF as well as respiratory parameters were recorded at the same time points at T-1, T1, and T2.

### Statistical analysis

The primary endpoint was the assessment of DTF of the right and left hemidiaphragms at T1 for patients with LUS ≥ 14 and those with LUS < 14. The secondary endpoints were as follows: (1) the right DE, TEE, and TEI at T1 in patients with LUS ≥ 14 and those with LUS < 14; (2) the correlation between the LUS and DTF at T1; and (3) the changes in the LUS and DTF from T-1 to T2 in patients with LUS ≥ 14.

Categorical variables were compared using a chi-square test or Fisher’s exact test and expressed as number and percentage. Quantitative variables were expressed as median and 25 to 75% interquartile range (IQR). Comparisons between the patients with LUS ≥ 14 and those with LUS < 14 were made using a Mann-Whitney *U* test. The changes in LUS from T-1 to T2 in patients with LUS ≥ 14 were compared using a Friedman-repeated measures analysis of variance on ranks followed by a Tukey post hoc analysis. The correlation between the LUS and DTF at T1 was determined by a Spearman correlation.

All analyses were performed using SigmaStat 3.5 (Systat Software Inc., Point Richmond, CA, USA) or SPSS 13.0 for Windows (SPSS Inc., Chicago, Il, USA). The statistical significance level was fixed at 0.05.

## Results

### Clinical characteristics

**Fig. 3:**
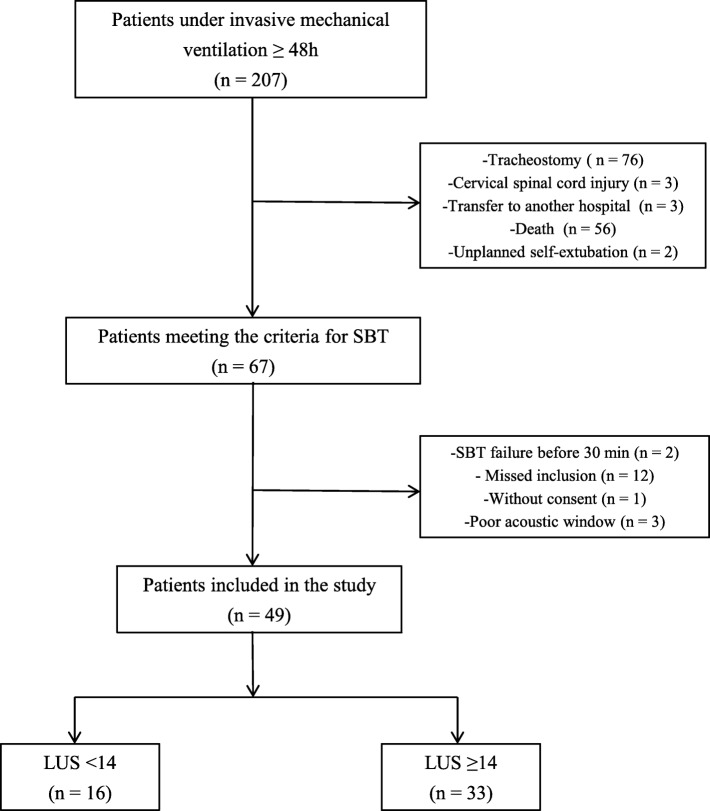
Flowchart of the study. SBT, spontaneous breathing trial; LUS, lung ultrasound aeration score

During the study period, a total of 207 patients underwent invasive mechanical ventilation ≥ 48 h, of which 67 patients underwent a SBT trial and 49 met the inclusion criteria. The flowchart is shown in Fig. 3.

Clinical characteristics of the patients are summarized in Table 1. At 30 min into the SBT, 16 patients had LUS < 14, with a median score of 10, and 33 patients had LUS ≥ 14, with a median score of 19. Age, severity scores at admission, causes of ICU admission, endotracheal size, and ventilator parameters before the SBT did not differ significantly between patients with LUS ≥ 14 and those with LUS < 14. PaO_2_/FiO_2_ was significantly lower in patients with LUS ≥ 14. The median duration of invasive mechanical ventilation before the SBT and the median total duration of mechanical ventilation were respectively 10 days, which were not different between the two groups.

**Table 1:** Clinical characteristics of the patients

	Overall (*n* = 49)	LUS < 14 *(n* = 16)	LUS ≥ 14 (*n* = 33)	*P* value
LUS	15 (11 to 21)	10 (8 to 11)	19 (15 to 22)	< 0.001
Age (year)	55 (41 to 67)	58 (43 to 71)	52 (41 to 66)	0.179
Sex (male)	33 (67%)	9 (56%)	24 (72%)	0.249
APACHE II at admission	13 (8 to 16)	15 (9 to 18)	13 (7 to 15)	0.375
SOFA at admission	6 (4 to 8)	5 (4 to 7)	6 (4 to 9)	0.390
Cause of admission (*n*)				0.239
Medical	26 (53%)	8 (50%)	18 (55%)	
Surgical	15 (31%)	7 (44%)	8 (24%)	
Trauma	8 (16%)	1 (6%)	7 (21%)	
ET size (mm)	7.5 (7.5 to 7.5)	7.5 (7 to 7.5)	7.5 (7.5 to 7.5)	0.418
Frequency-T-1 (breaths/min)	17 (15 to 21)	16 (14 to 22)	18 (15 to 21)	0.571
Pressure support level T-1 (cmH_2_O)	8 (8 to 10)	8 (8 to 10)	8 (8 to 9)	0.834
PEEP T-1 (cmH_2_O)	5 (5 to 6)	5 (5 to 6)	5 (5 to 6)	0.927
V_T_ T-1 (ml)	495 (409 to 571)	450 (418 to 591)	514 (403 to 563)	0.981
FiO_2_ (%) T-1	40 (35 to 40)	40 (35 to 40)	40 (34 to 45)	0.237
PaO_2_/FiO_2_ T-1 (mmHg)	244 (213 to 278	279 (236 to 339)	238 (208 to 205)	0.016
Duration of MV before SBT (day)	10 (4 to 15)	9 (3 to 12)	10 (4 to 16)	0.412
Total duration of MV (day)	10 (5 to 17)	9 (4 to 12)	10 (6 to 18)	0.197

Data are expressed as median and 25–75% IQR

*LUS* lung ultrasound aeration score, *APACHE* Acute Physiology and Chronic Health Evaluation, *SOFA* Sequential Organ Failure Assessment, *MV* mechanical ventilation, *SBT* spontaneous breathing trial, *ET* endotracheal tube, *T-1* during mechanical ventilation 1 h before SBT, *PEEP* positive end-expiratory pressure, *V*_*T*_ tidal volume, *Frequency* respiratory rate

### Diaphragmatic function at T1 and the correlation between DTF and LUS

**Fig. 4:**
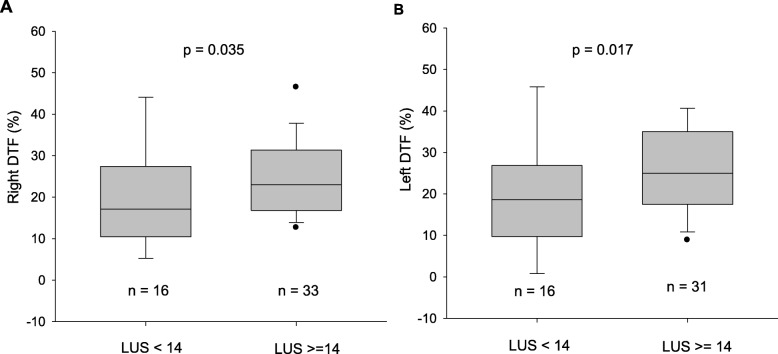
Comparison of the DTF between patients with LUS < 14 and those with LUSs ≥ 14. Measurements were performed after 30 min of a SBT. DTF, diaphragmatic thickening fraction; LUS, lung ultrasound aeration score; **a** right DTF; **b** left DTF. The left DTF was not visualized in 2 patients

In all patients, the DTF did not differ between the right and left hemidiaphragms [21.8% (14.5 to 29.7)% vs. 23.4% (16.5 to 31.5)%, *p* = 0.561]. Compared to the patients with LUS < 14, a significant increase in the DTF of the right and left hemidiaphragms was observed in the patients with LUS ≥ 14 (Fig. 4 and Table 2). The right DE, TEI, TEE, PaO_2_, PaCO_2_, and respiratory rate did not differ between the patients with LUS ≥ 14 and those with LUS < 14 (Table 2).

**Table 2:** Right diaphragmatic function and respiratory parameters at T1 in patients with LUS ≥ 14 and those with LUS < 14

	Overall (*n* = 49)	LUS < 14 (*n* = 16)	LUS ≥ 14 (*n* = 33)	*P* value
R-DE (cm)	1.2 (0.9 to 1.7)	1.5 (1.0 to 1.7)	1.2 (0.9 to 1.5)	0.495
R-TEI (mm)	1.9 (1.6 to 2.3)	1.9 (1.5 to 2.2)	1.9 (1.7 to 2.5)	0.502
R-TEE (mm)	1.6 (1.3 to 1.9)	1.6 (1.3 to 1.8)	1.6 (1.4 to 1.9)	0.669
R-DTF (%)	21.8 (14.5 to 29.7)	14.8 (10.2 to 27.0)	22.2 (17.1 to 30.9)	0.035
PaO_2_ (mmHg)	97 (78 to 136)	112 (79 to 144)	96 (76 to 118)	0.501
PaCO_2_ (mmHg)	37 (34to 40)	37 (32 to 41)	37 (35 to 39.0)	0.991
Frequency (breaths/min)	22 (18 to 25)	23 (19 to 25)	20 (18 to 25)	0.396

Data are expressed as median and 25–75% IQR. T1 = 30 min after the start of spontaneous breathing trial

*LUS* lung ultrasound aeration score, *R* right, *DE* diaphragm excursion, *TEI* diaphragm thickness at end-inspiration, *TEE* diaphragm thickness end-expiration, *DTF* diaphragmatic thickening fraction, *Frequency* respiratory rate

**Fig. 5:**
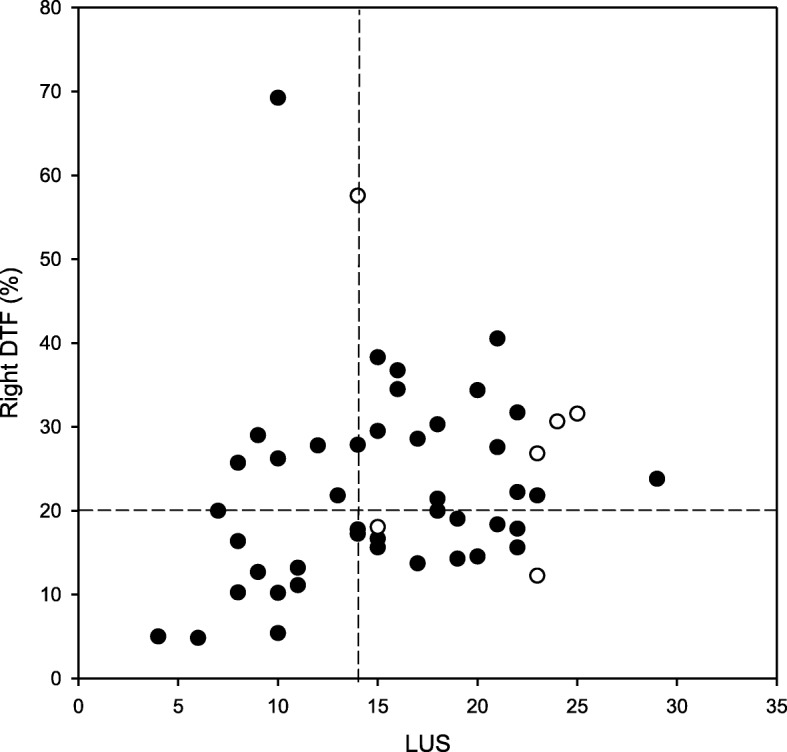
Scatter plot between lung aeration score (LUS) and right diaphragmatic thickening fraction (DTF) assessed by ultrasound. Filled circles indicate the patients successfully weaned from mechanical ventilation; open circles indicate the patients with weaning failure. The vertical dashed line indicates LUS of 14; the horizontal dash line indicates DTF of 20%. LUS and DTF were assessed at 30-min after a spontaneous breathing trial

Lung aeration did not differ between the right and left lungs [right LUS, 9 (5 to 10) vs. left LUS, 8 (6 to 10), *p* = 0.145]. A moderate but significant positive correlation was found between the LUS and DTF (Rho = 0.3, *p* = 0.014, Fig. 5).

### Lung aeration and DTF changes from T-1 to T2 in patients with LUS ≥ 14

Of the 33 patients with LUS ≥ 14 who successfully passed the 30-min SBT, 1 patient failed after 120 min of SBT requiring reconnection to the ventilator, and 5 patients were reintubated and/or tracheostomized. The individual values of LUS and right DTF at T1 of the 6 patients are specified in Fig. 5.

**Fig. 6:**
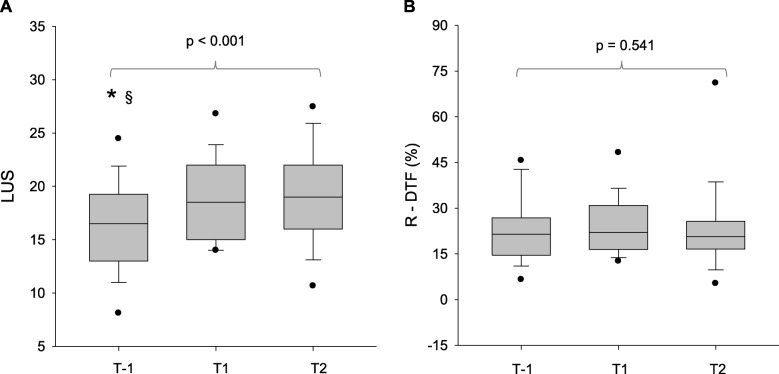
Evolution of the LUS (**a**) and right DTF (**b**) from T-1 to T2. T-1, during mechanical ventilation 1 h before the spontaneous breathing trial (SBT); T1 = 30 min after the start of the SBT; T2 = 120 min after the start of the SBT (T2). * = T-1 versus T1, *p* < 0.05; § = T-1 versus T2, *p* < 0.05 using a Friedman repeated measures analysis of variance on ranks followed by a Tukey post hoc analysis

As shown in Fig. 6, a significant increase in the LUS from 17 to 19 (median values) was observed from T-1 to T1, while no changes between T1 and T2 were observed. The DTF remained stable from T-1 to T2.

## Discussion

The results of this study showed that, after achieving 30 min of SBT, the diaphragmatic contractility estimated by the DTF was significantly higher in patients with high lung aeration loss, defined as a LUS ≥ 14, than in patients with low lung aeration loss (LUS < 14). The DTF was moderately and positively correlated with the degree of aeration loss. In patients with LUS ≥ 14, the LUS was significantly higher after 30 min of SBT than during mechanical ventilation, indicating lung derecruitment, while the DTF remained stable before and after the patient was disconnected from the ventilator. No other changes were found for the LUS and DTF after 120 min of SBT.

Transthoracic lung ultrasound is increasingly used in the ICU for the bedside assessment of lung aeration. The LUS assessed by ultrasound has been shown to correlate with the volume of gas measured by computed tomography [20, 21]. In a previously published study, Sommer et al. demonstrated that a LUS greater than 14 was associated with a high risk of developing post-extubation distress [4]. However, an inconclusive range between 12 and 17 has been reported, and diaphragmatic function, another factor influencing weaning failure [9], has not been studied. Diaphragm is the main inspiratory muscle. The contraction of the diaphragmatic muscle causes it to shorten essentially at the area of apposition, generating a tidal volume by a decrease in intra-thoracic pressure. DTF has been shown to correlate with transdiaphragmatic pressure, which reflects the work involved in breathing [22]. It can be hypothesized that in patients with adequate diaphragmatic function, the diaphragmatic contraction during a SBT may contribute to increased inspired lung volume, which would promote successful weaning in patients with reduced lung mechanics characterized by high lung aeration loss. To our knowledge, our study is the first to assess the relationship between lung aeration and diaphragmatic function during a weaning process with ultrasound.

In the present study, we included only patients who successfully completed the first 30-min SBT aimed at reducing the number of patients with a difficult weaning history [3], as a decrease in diaphragmatic function was frequently observed in these patients [23]. Our results showed that the DTF after 30 min of SBT was significantly higher in patients with LUS ≥ 14 than in those with LUS < 14. This result suggested that the patients with high lung aeration loss increased their breathing effort to compensate the lung volume loss. Similarly, the diaphragmatic workload was decreased when the patients experienced normal lung aeration or moderate lung aeration loss [14, 24]. It should be noted that LUS is a semi-quantitative lung aeration score that does not quantify the tidal lung volume changes during a SBT. Interestingly, our results revealed a moderate but positive correlation between the LUS and DTF, which suggests that diaphragm contractility increases when the degree of lung aeration loss is high.

In previously published studies, inspiratory diaphragmatic thickness was measured at either end-inspiration during quiet breathing or at TLC [9, 12, 25]. As a result, the reported DTF may be different depending on the method used. In healthy subjects, the reported lower limit of the DTF was 20% [26, 27]. It has also been reported that DTF > 20% was associated with SBT success [9, 25, 28, 29]. The median DTF for the right and left hemidiaphragms in our study were respectively 15% and 19% in patients with LUS < 14, and 22% and 25% in those with LUS ≥ 14. In addition to the reduced workload in patients with low lung aeration loss, diaphragm dysfunction in some patients could not be ruled out because it is known that diaphragmatic weakness can occur rapidly during mechanical ventilation [30]. Despite this low DTF, all patients with LUS < 14 were successfully weaned from mechanical ventilation. Conversely, 6 out of the 33 patients with LUS ≥ 14 failed the weaning process: 4 patients had a right DTF > 20% and 4 had severe lung aeration loss with LUS between 23 and 25. In a patient admitted for severe acute pancreatitis, the LUS score was 15; however, he had diaphragmatic dysfunction with low DTF in the right and left hemidiaphragms as well as decreased DE. Some patients with LUS ≥ 14 and right DTF ≤ 20% were weaned off mechanical ventilation. When considering the DTF of the right and left hemidiaphragms, 4 patients had DTF on both sides < 20%, and 2 failed to be weaned from mechanical ventilation. These results indicate that the mechanisms involved in the weaning process are complex; neither diaphragmatic function nor lung aeration alone could predict precisely weaning failure. Very likely, in these patients, the addition of other compensatory indices such as rapid shallow breathing index and accessory muscle use would also be helpful to better assess patient’s compensatory capacity for lung volume loss.

In addition, the absence of a DTF difference between the right and left hemi-diaphragm indicates that, in most patients, measurement of the right hemidiaphragm can be sufficient to evaluate diaphragmatic function, especially since the left hemidiaphragm is more difficult to visualize [12].

Our study confirmed the results of Soummer et al. who showed alveolar derecruitment in some patients with LUS ≥ 14 during the SBT [4]. In addition, the absence of changes in the LUS and DTF between T1 and T2 justifies the SBT efficacy at 30 min compared to that at 120 min in terms of lung aeration and diaphragmatic function assessment [15].

Several limitations of the study need to be discussed. First, the diaphragm is very thin, and small measurement errors may result in an overestimation or underestimation of the thickness and thickening fraction. A number of studies have addressed the concern of consistency and have shown acceptable results in terms of inter- and intraobserver variability [12, 31]. In our study, to increase measurement accuracy, the probe placement site was marked for each evaluation, and the probe was always positioned at the same site for further measurements. In addition, 3 measurements were taken and averaged to reduce intraobserver variability. Interobserver variability was not assessed. Second, the cutoff LUS value of 14 to differentiate high and low lung aeration remains debatable. Although good agreement between observers has been reported in previously published studies [21, 32], it is sometimes not easy to distinguish between the B1 and B2 lines in some patients, which could lead to a difference of 1 or 2 points in the evaluation of the LUS between 2 operators. In our study, the interobserver variability was assessed for 50 LUS evaluations. An average difference of 1 ± 2 points in the LUS was found between the 2 operators. Third, inspired and expired lung volumes were not measured in this study. As mentioned above, the degree of lung aeration loss assessed by the LUS reflected the severity of lung injury, but the LUS was unable to measure tidal lung volume changes during the SBT. Therefore, an increased DTF in patients with LUS ≥ 14 vs. patients with LUS < 14 suggest additional breathing efforts by the patients, and the amplitude of variation in the tidal volume resulting from the contraction of the diaphragm remains unknown. As it has been well demonstrated that the inspired/expired volume correlates linearly with the DTF in healthy subjects and in patients during the weaning process [27, 28], it can be inferred that in some patients with high lung aeration loss, an increase in diaphragmatic contractility during a SBT would contribute to an increase in tidal lung volume, which would partly explain the successful SBT and weaning. However, if the additional breathing effort could not compensate lung volume loss, this unnecessary increase of work of breath could lead to weaning failure. Finally, this was a single-center study with a small sample size. The results of this study can be considered pilot explorations that deserve further investigation.

## Conclusions

During a 30-min SBT, the diaphragmatic contraction acts differently according to the degree of lung aeration loss. Diaphragmatic contraction was significantly greater in patients with high lung aeration loss than in those with low lung aeration loss. Further large-scale studies with physiological index measurements are needed to accurately assess the compensatory capacity of the diaphragm for volume loss in patients with LUS ≥ 14; and to evaluate the benefits of a combined evaluation of lung aeration and diaphragmatic function by ultrasound to predict successful weaning of patients from mechanical ventilation.

## Data Availability

The datasets used and/or analyzed during the current study are available from the corresponding author on reasonable request.

## Abbreviations

DE: Diaphragmatic excursion
DTF: Diaphragmatic thickening fraction
FRC: Functional residual capacity
ICU: Intensive Care Unit
IQR: Interquartile range
LUS: Lung aeration ultrasound score
SBT: Spontaneous breathing trial
T1: 30 min after the start of SBT
T-1: During mechanical ventilation 1 h before the SBT
T2: 120 min after the start of SBT
TEE: Diaphragmatic thickness at end-expiration
TEI: Diaphragmatic thickness at end-inspiration
TLC: Total lung capacity
